# The complete chloroplast genome sequence of *Annamocarya sinensis* (Juglandaceae), an Endangered species endemic to Yunnan Province, China

**DOI:** 10.1080/23802359.2020.1756477

**Published:** 2020-05-12

**Authors:** Yuman Ji, Wanjie Zhang, Dan Li, Lixin Shen

**Affiliations:** a APFNet Kunming Training Center, Southwest Forestry University, Kunming, China; b Faculty of Biodiversity of Conservation, Southwest Forestry University, Kunming, China

**Keywords:** *Annamocarya sinensis*, Endangered species, chloroplast genome, phylogenetic analysis

## Abstract

*Annamocarya sinensis*, a plant species with extremely small populations endemic to Xichou county of Yunnan province, has been classified as a national second-class protected wild plant. In this study, we assembled its complete chloroplast genome. The total genome size of *A. sinensis* was 158,484 bp in length, containing a large single-copy region of 89,871 bp, a small single-copy region of 20,558 bp, and a pair of inverted repeat regions of 24,029 bp. The all GC content of *A. sinensis* chloroplast genome was 36.2%. It encodes a total of 114 unique genes, including 79 protein-coding genes, 31 tRNA genes, and four rRNA genes. Eleven genes contain a single intron, and three genes have two introns. Phylogenetic analysis results strongly supported that *Annamocarya sinensis* was closely related to *Carya kweichowensis*.

*Annamocarya sinensis* is an Endangered species that needs urgent conservation action. There is only one species in the genus *Annamocarya* which is *A. sinensis*. In China, it is mainly distributed in Yunnan, Guizhou, and Guangxi Province in the form of small groves or isolated individuals (Long 2002). In 1985, the Environmental Protection Commission of the State Council listed *A. sinensis* as the first batch of rare and endangered key protected tree species in Yunnan Province (Zhang et al. 2013). It had been classified as a national second-class protected wild plant in the Information System of Chinese Rare and Endangered Plants (ISCRPE) (http://www.iplant.cn/rep/prot/Annamocaryasinensis). Now, it is extremely endangered and on the verge of extinction. Therefore, it is necessary to protect the germplasm resources of *A. sinensis*.

Chloroplast genome is exceptionally conserved in gene content and organization, providing sufficient resources for genome-wide evolutionary studies and has demonstrated the potential to resolve phylogenetic relationships at different taxonomic levels, and understand structure and functional evolution, by using the whole chloroplast genome sequences (Jansen et al. 2007; Moore et al. 2010). So far, the chloroplast genome such as *Carya kweichowensis* within the family of Juglandaceae has been reported, but the Chloroplast genome of *A. sinensis* has not been reported. Now, we reported the complete chloroplast genome sequence of *A. sinensis* based on the next-generation sequencing, and the annotated genomic sequence was submitted to GenBank under accession number MN911165.

The fresh leaves of *Annamocarya sinensis* were collected from Xichou county of Yunnan province. Total genome DNA was extracted with the Ezup plant genomic DNA prep kit (Sangon Biotech, Shanghai, China). The voucher specimens of *A. sinensis* were deposited at the herbarium of Southwest Forestry University (accession number: SWFU-YAB-H-0160), and DNA samples were properly stored at Key Laboratory of State Forestry Administration on Biodiversity Conservation in Southwest China, Southwest Forestry University, Kunming, China. Total DNA was used to generate libraries with an average insert size of 350 bp, which were sequenced using the Illumina HiSeq X platform. Approximately, 14.0 GB of raw data were generated with 150 bp paired-end read lengths. Then, the raw data were used to assemble the complete cp genome using GetOrganelle software (Jin et al. 2018) with *Juglans nigra* as the reference. Genome annotation was performed with the program Geneious R8 (Biomatters Ltd, Auckland, New Zealand) by comparing the sequences with the cp genome of *Juglans nigra*. The tRNA genes were further confirmed through online tRNAscan-SE web servers (Schattner et al. 2005). A gene map of the annotated *A. sinensis* cp genome was drawn by OGdraw online (Lohse et al. 2013).

The chloroplast genome of *A. sinensis* exhibited a general quadripartite structure of plants, with two reverse repeated regions (IRa and IRb) of 24,029 bp in length. The repeat regions divided the genome into two single-copy regions, SSC and LSC with 20,555 bp and 89,871 bp, respectively. The GC contents of the LSC, SSC, and IR regions individually, and of the cp genome as a whole, are 33.8, 34.7, 43.0, and 36.2%, respectively. It encodes a total of 114 unique genes, of which 15 are duplicated in the IR regions. Out of the 114 genes, there are 79 protein-coding genes, 31 tRNA genes, and 4 rRNA genes. Fourteen genes contained introns, 11 (seven protein-coding and four tRNA genes) of which contained one intron and three of which (*rps12*, *ycf3*, and *clpP*) contained two introns.

To confirm the phylogenetic location of *A. sinensis* within the family of Juglandaceae, a total of 13 complete cp genomes of Juglandaceae were obtained from GenBank, and *Platycarya strobilacea* in the genus of *Platycarya* of Juglandaceae family was used as out-group. The 14 complete chloroplast sequences were aligned by the MAFFT version 7 software (Katoh and Standley 2013). Phylogenetic analysis was conducted based on maximum likelihood (ML) analyses implemented in IQ-TREE 1.5.5 (Nguyen et al., 2015) under the TVM + F+R2 nucleotide substitution model, which was selected by ModelFinder (Kalyaanamoorthy et al. 2017). Support for the inferred ML tree was inferred by bootstrapping with 1000 replicates. Phylogenetic analysis results strongly supported that *A. sinensis* was closely related to *Carya kweichowensis* (Figure 1). The chloroplast genome of *A. sinensis* will provide useful genetic information for further study on genetic diversity and conservation of Juglandaceae species.

**Figure 1.**
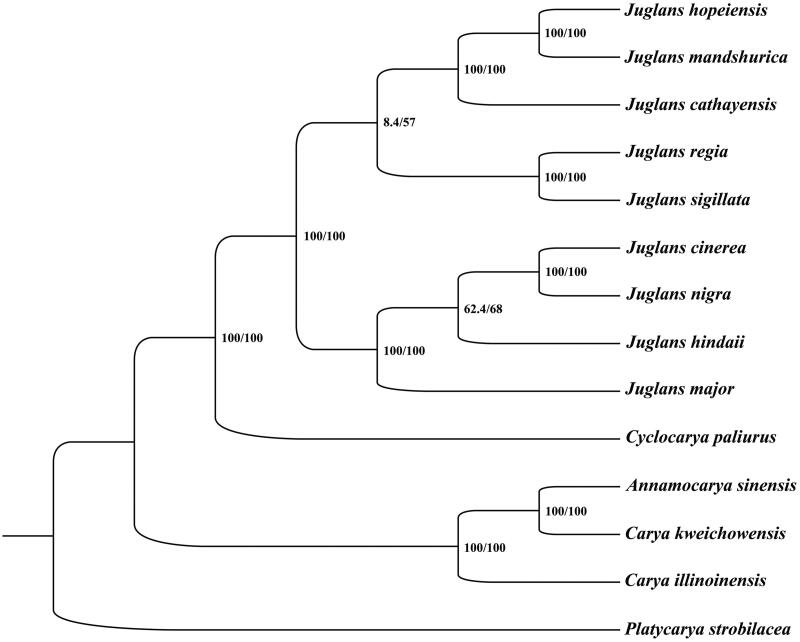
Phylogenetic relationships among 14 complete chloroplast genomes of Juglandaceae. Bootstrap support values are given at the nodes. Chloroplast genome accession number used in this phylogeny analysis: *Juglans hopeiensis*: KX671977; *Juglans mandshurica*: MF167461; *Juglans cathayensis:* MF167457; *Juglans regia*: MF167463; *Juglans sigillata*: MF167465; *Juglans cinerea*: MF167458; *Juglans nigra*: MF167462; *Juglans hindsii*: MF167459; *Juglans major*: MF167460; *Cyclocarya paliurus*: KY246947; *Annamocarya sinensis*: MN911165 (the sample in this study); *Carya kweichowensis*: MH121170; *Carya illinoinensis*: MH909599; *Platycarya strobilacea*: KX868670.

## Data Availability

The data that support the findings of this study are openly available in GenBank of NCBI at https://www.ncbi.nlm.nih.gov, reference number MN911165.
